# A Rare Collision in Thyroid and Lymph Node: A Case Report and Review of Literature

**DOI:** 10.7759/cureus.86190

**Published:** 2025-06-17

**Authors:** Gowtham K V, Suhaildeen Kajamohideen, Arthi M, Leena D Joseph, Ilango Parthasarathy

**Affiliations:** 1 Surgical Oncology, Sri Ramachandra Institute of Higher Education and Research, Chennai, IND; 2 Pathology, Sri Ramachandra Institute of Higher Education and Research, Chennai, IND

**Keywords:** braf mutation, carcinoembryonic antigen, familial medullary thyroid cancer, medullary thyroid carcinoma, men syndrome, papillary thyroid carcinoma, ret protooncogene, serum calcitonin, synchronous tumors

## Abstract

Synchronous papillary and medullary thyroid carcinomas are exceptionally rare. This case report describes a 13-year-old girl with familial medullary thyroid cancer linked to germline rearranged during transfection (RET) proto-oncogene mutation, with a high risk of early onset and aggressive disease, who underwent total thyroidectomy with prophylactic neck dissection. Histopathology revealed distinct papillary and medullary thyroid cancers in separate thyroid lobes, which was a type IV synchronous tumor, with one lymph node showing metastatic deposits from both tumors. This case highlights the rare occurrence, diagnostic challenges of such dual malignancies, emphasizing the need for genetic testing and vigilant long-term follow-up.

## Introduction

Thyroid cancers are the most prevalent endocrine malignancies. Among thyroid cancers, differentiated thyroid cancers (DTC) originating from thyroid follicular cells comprise 85% of all cases, with papillary thyroid cancer (PTC) being the most frequent. In contrast, medullary thyroid cancer (MTC) is a rare neuroendocrine tumor, accounting for approximately 4% of thyroid malignancies [1]. It is distinct from other thyroid malignancies due to its neuroendocrine origin and unique biological behavior. Sporadic MTC constitutes 75% to 80% of cases, with hereditary MTC making up the remaining 20% to 25%, and it is associated with an autosomal dominant germline mutation in the rearranged during transfection (RET) proto-oncogene and often manifests as part of multiple endocrine neoplasia type 2 (MEN 2). The coexistence of medullary and papillary thyroid cancer arising from different cellular lineages is exceptionally rare, with an occurrence of 0.2% to 1.2 % of thyroid malignancies [2,3]. In such occurrences, MTC exhibits aggressive behavior, whereas PTC typically has a more indolent course, with a 97.5% overall five-year relative survival [4]. This report presents a case of familial medullary thyroid cancer (FMTC), with an incidental occurrence of PTC coexisting with MTC on final histopathological analysis. The unexpected presence of PTC raises the possibility of additional genetic mutations or shared molecular pathways in patients with FMTC, contributing to this rare synchronous phenomenon.

## Case presentation

**Figure 1:**
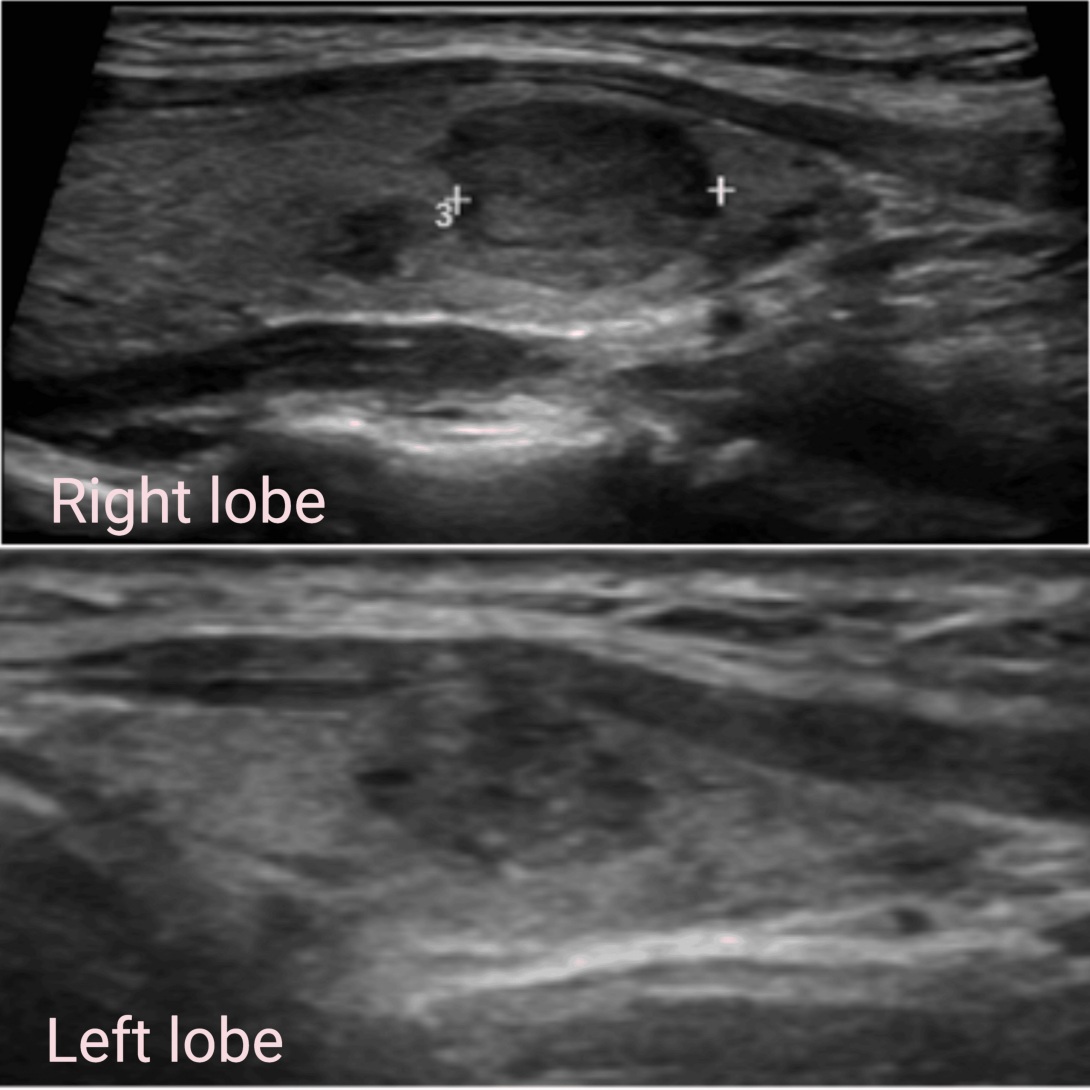
Ultrasound showing nodules in right and left lobe of thyroid

This case report is about a 13-year-old girl who was evaluated after her 40-year-old father was diagnosed with MEN 2B-associated MTC. The father did not have a known family history of medullary thyroid carcinoma (MTC) or any other types of cancer. He had undergone total thyroidectomy with bilateral neck dissection and bilateral adrenalectomy for bilateral pheochromocytoma at the age of 38 years. Genetic testing by next-generation sequencing (NGS) confirmed a RET c.1900T>C (p.Cys634Arg) proto-oncogene germline mutation at codon 634 in Exon 11 in the father, which is a well-established pathogenic variant associated with FMTC syndrome. This mutation is classified as pathogenic according to the American College of Medical Genetics and Genomics (ACMG) criteria. MTC associated with the RET proto-oncogene mutation is inherited in an autosomal dominant pattern, with a chance of passing the mutation to the offspring. The family was counseled about the autosomal dominant inheritance of the RET mutation, risk of developing MTC and pheochromocytoma, recommendations for genetic testing in first-degree relatives, and the importance of early detection and prophylactic surgery. Both of his children were screened, revealing multiple nodules in both lobes of the thyroid with subcentimetric nodes with maintained fatty hilum in ultrasound neck with raised serum tumor markers. The elder son had a Bethesda category VI thyroid nodule, positive for MTC with no evidence for pheochromocytoma on evaluation. He underwent total thyroidectomy with prophylactic central compartment and bilateral level II to V neck dissection. Ultrasound neck in his daughter showed multiple nodules in both lobes of thyroid, largest nodule was 1.2 x 1 x 0.9 cm (ACR TIRADS 5) in the right lobe and 0.8 x 0.8 x 0.5 cm (ACR TIRADS 4) in the left lobe with subcentimetric nodes in central and lateral neck with preserved fatty hilum (Figure 1).

Fine-needle aspiration cytology (FNAC) of a TIRADS 5 nodule in the right lobe was Bethesda category III- Atypia of undetermined significance. Her serum calcitonin and CEA were elevated (Table 1).

**Table 1:** Serum tumor markers

Serum markers	Result	Reference value
Serum calcitonin	175 pg/ml	0-11.50 pg/ml
Serum carcinoembryonic antigen (CEA)	12 ng/ml	Non smoker : <3.8 ng/ml ; Smoker : <5.5 ng/ml

Her 24-hour urinary and plasma metanephrines, normetanephrines, and ultrasound abdomen were normal. As per our institute's multidisciplinary tumor board recommendation, she underwent total thyroidectomy with prophylactic central compartment and bilateral level II to V neck dissection. Intraoperative period and postoperative recovery were uneventful.

Histopathology

**Figure 2:**
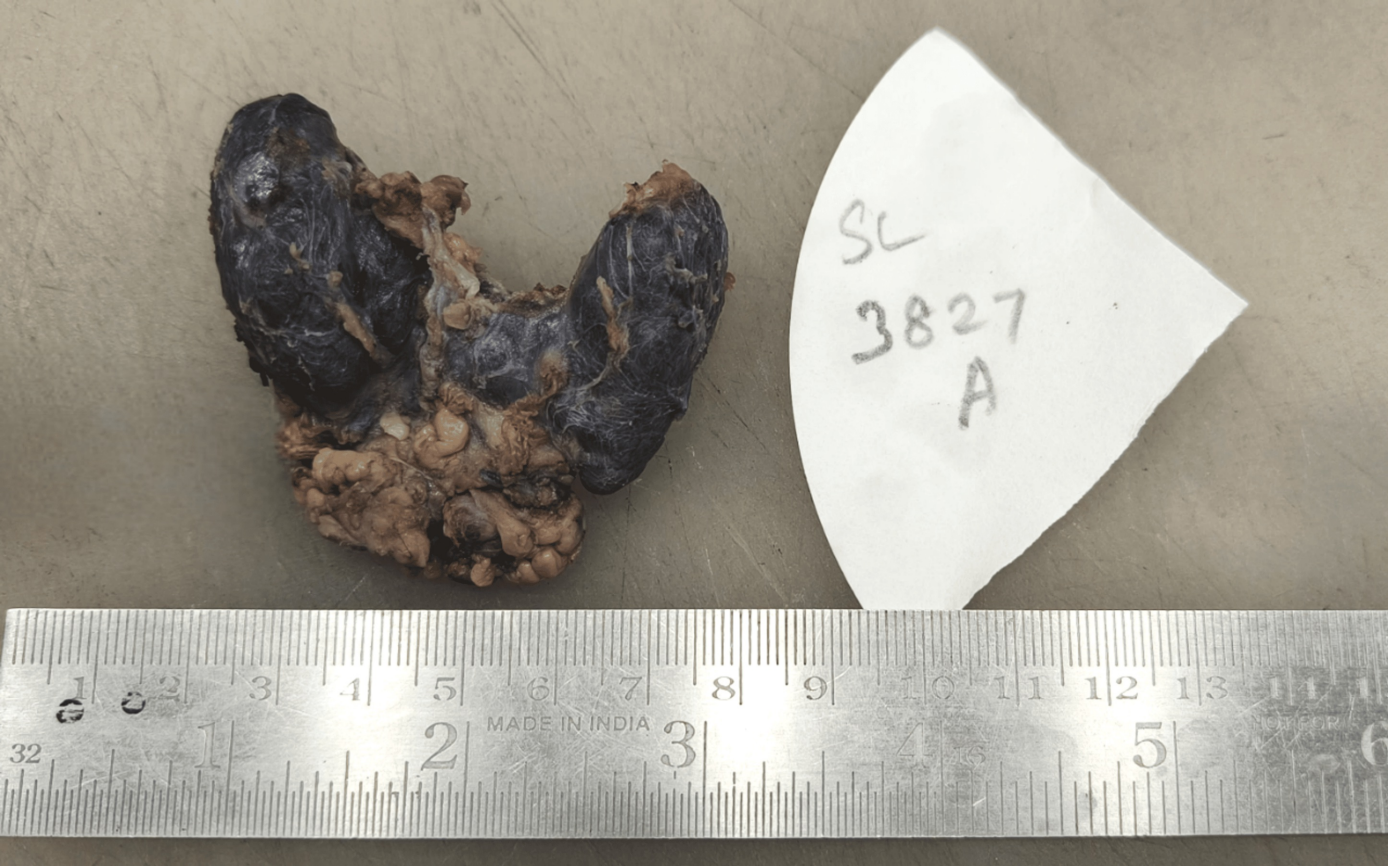
Total thyroidectomy gross specimen

**Figure 3:**
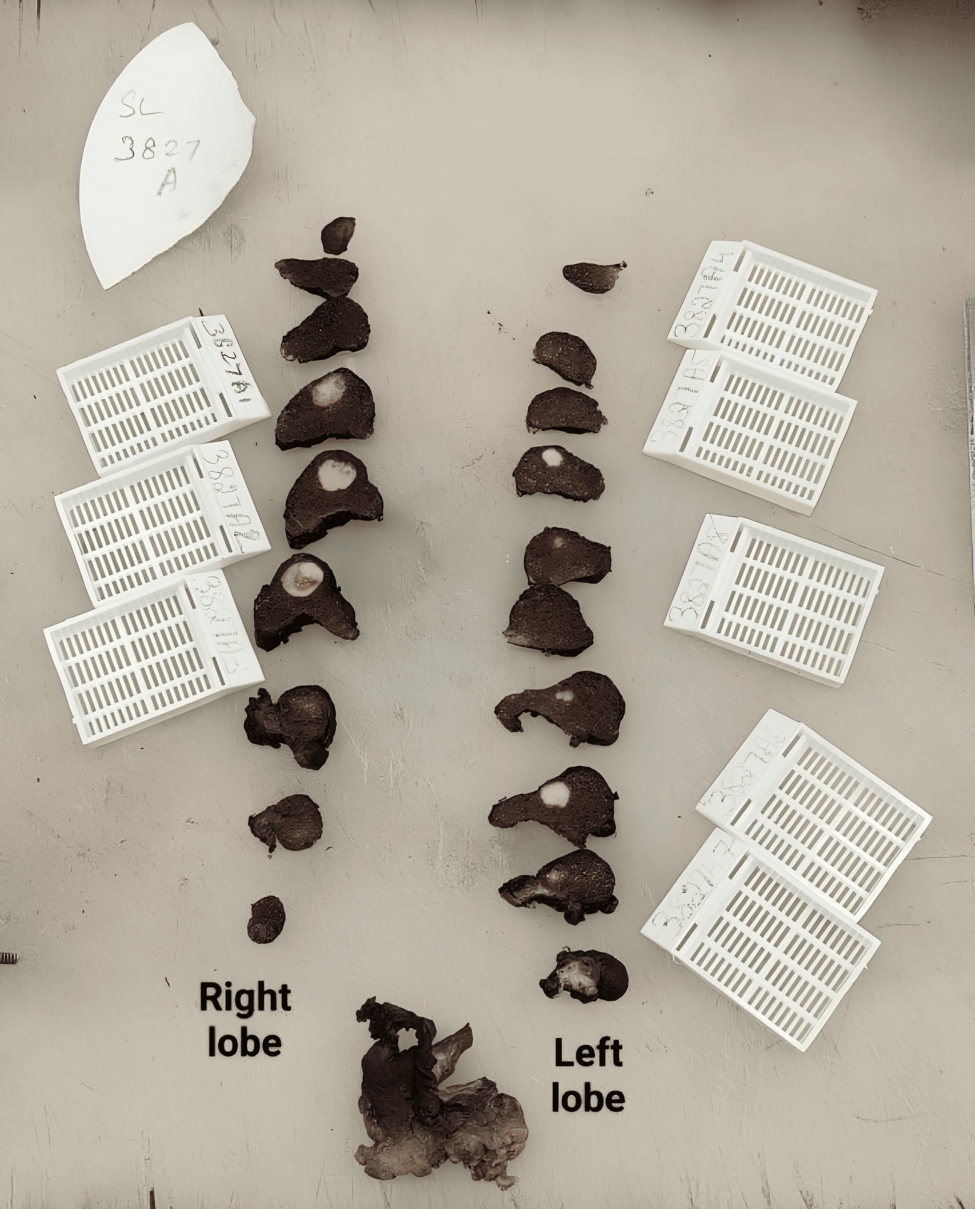
Cut section showing lesion in both lobes of thyroid

**Figure 4:**
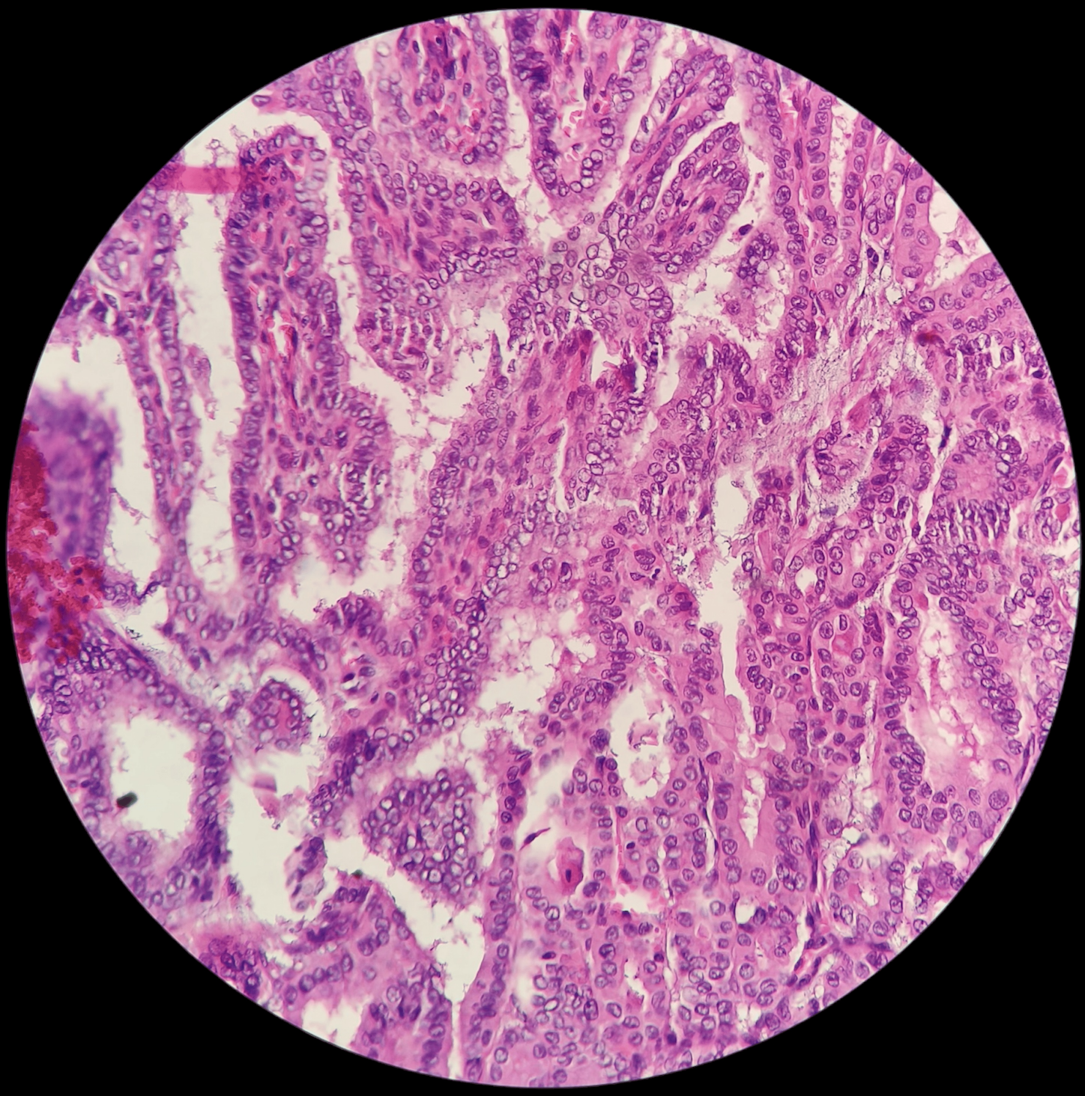
H&E 400X showing papillary carcinoma thyroid with cells arranged in papillary architecture and characteristic nuclear features

**Figure 5:**
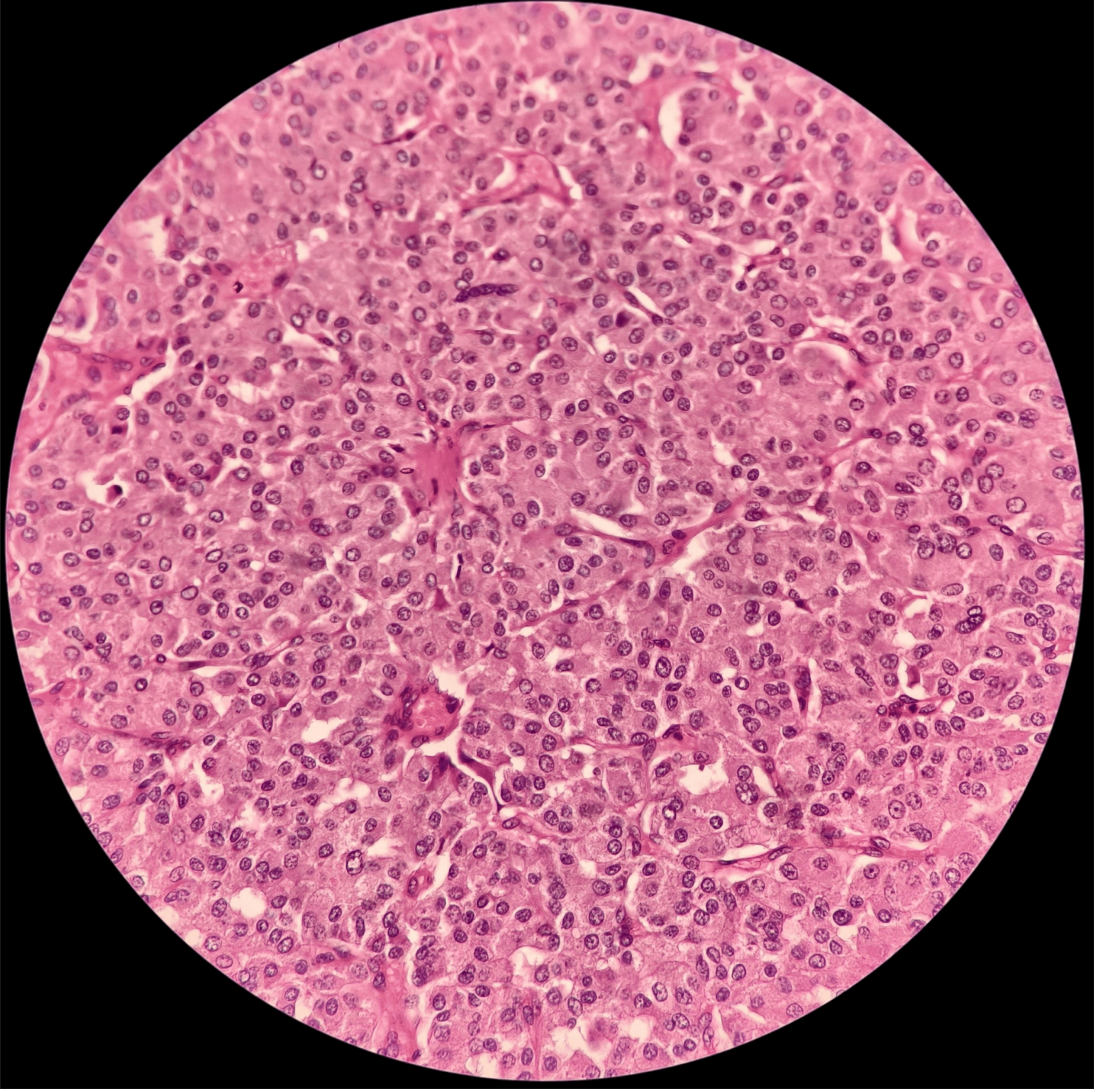
H&E 400X medullary carcinoma thyroid showing cells arranged in sheets with plasmacytoid features and amyloid stroma

**Figure 6:**
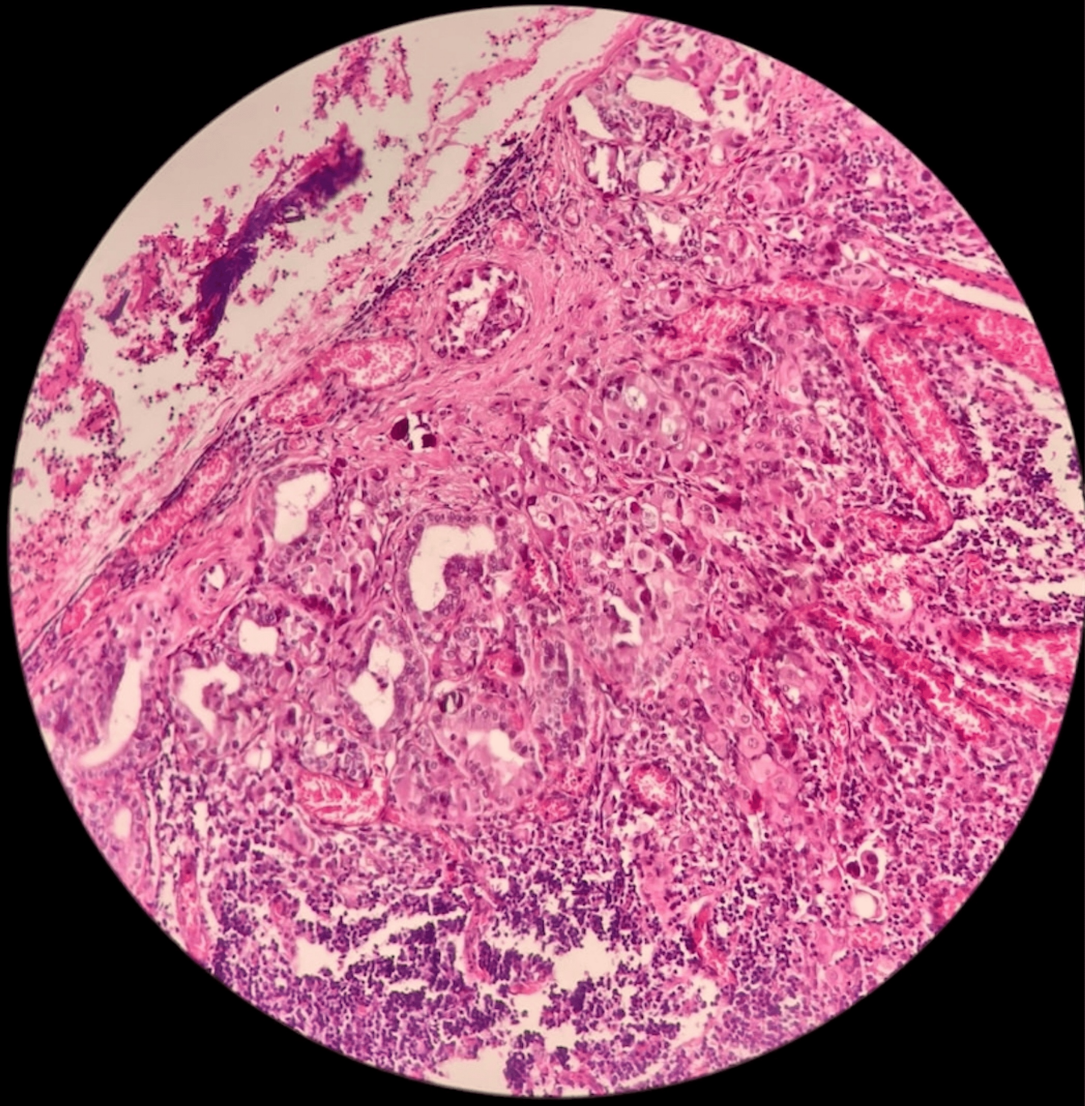
H&E 200X showing lymph node with metastatic deposit from both medullary and papillary carcinoma thyroid

On gross, the total thyroidectomy specimen had multiple nodules in both lobes. The right lobe of the thyroid had a 1.5 x 1 x 1 cm solid lesion, and the left lobe had two lesions of size 0.4 x 0.4 x 0.4 cm near the superior pole and 0.6 x 0.6 x 0.6 cm close to the isthmus, respectively (Figures 2, 3). A total of 73 nodes were grossed, and the largest node was of size 2.5 x 1.5 x 0.5 cm in the left level II region. Microscopy revealed two distinct histologies in the total thyroidectomy specimen. Left lobe of thyroid had two solid lesions of size 0.6 x 0.6 x 0.6 cm with a histology of papillary carcinoma, classic subtype and 0.4 x 0.4 x 0.4 cm lesion with a histology of medullary carcinoma with amyloid deposition and right lobe had one lesion of size 1.5 x 1 x 1cm with medullary histology (Figures 4, 5). Two central compartment lymph nodes were positive out of 73 nodes. One of the lymph nodes showed tumor deposits from both MTC and PTC (Figure 6). Surgical margins were clear, and the rest of the thyroid parenchyma was reported normal.

**Figure 7:**
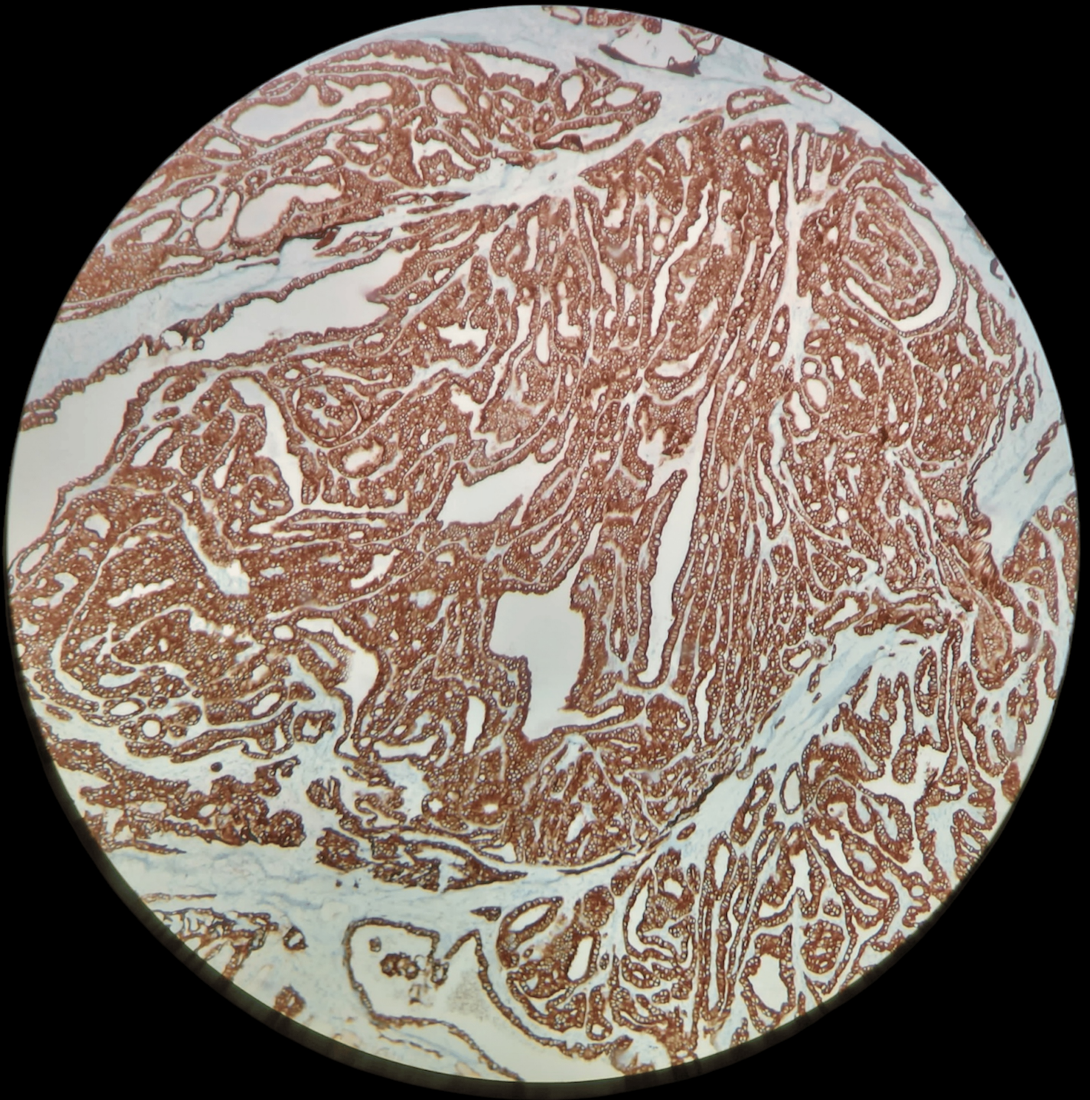
IHC 200X - CK 19 showing strong positivity in papillary carcinoma thyroid

**Figure 8:**
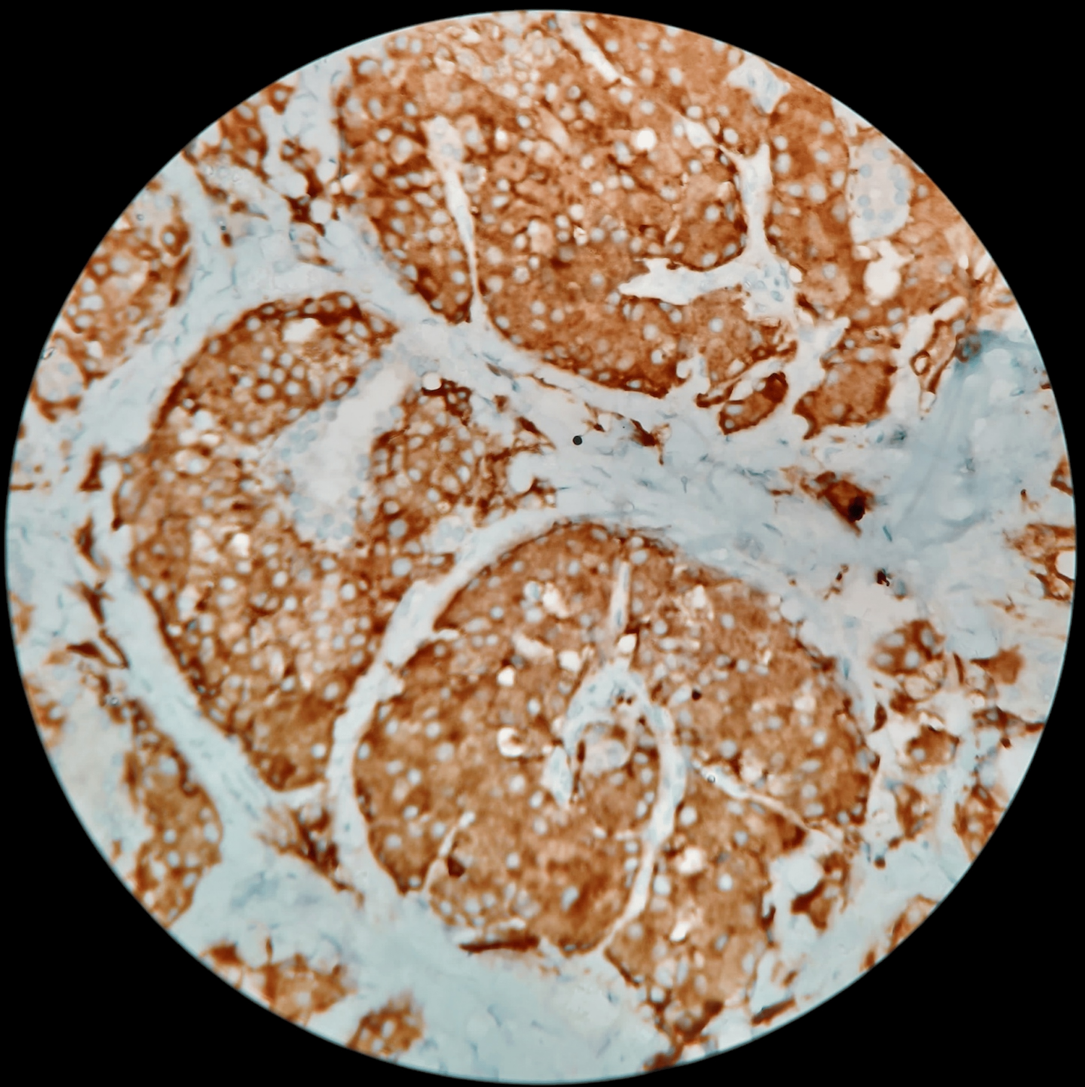
IHC 200X - CD 56 showing positivity in medullary carcinoma thyroid

Further on immunohistochemistry (IHC), CK19 was strongly positive in classic papillary thyroid carcinoma, medullary thyroid carcinoma, and tumor deposits in lymph nodes (Figure 7). CD56 had faint positivity in MTC and lymph node deposits (Figure 8). Synaptophysin was positive in MTC, and metastatic deposits were found in the node. There were no features of lymphatic and angioinvasion and no extrathyroidal extension.

It was a mpT1b N1a stage tumor as per TNM (AJCC 8th edition). The patient was counseled for radioactive iodine ablation therapy.

## Discussion

Papillary and medullary thyroid cancers originate from distinct embryonic cell lines. PTC arises from endoderm-derived follicular cells, whereas MTC originates from parafollicular C cells of the ultimobranchial body of neural crest. MTC is rare and aggressive when compared to PTC. PTC is driven by several genetic mutations and rearrangements that include BRAF and RAS mutations, TERT promoter mutations, RET /PTC rearrangements, and NTRK1 fusions, which play a crucial role in its pathogenesis and clinical behavior [5]. The synchronous occurrence of both PTC and MTC in the same thyroid with a family history of medullary thyroid cancer is rare, accounting for approximately 0.2% to 1.2% of all thyroid malignancies [2,3]. Among these, cases occurring in the setting of familial or hereditary MTC, such as MEN2-associated RET mutations, are even more uncommon, with only isolated case reports and small series described in the literature. They are typically discovered incidentally during histopathological evaluation after thyroidectomy, as their coexistence is often not apparent on routine preoperative evaluation.

Synchronous PTC-MTC tumors have been classified into four subtypes. Type I (true mixed tumors) consists of medullary and papillary thyroid carcinomas that are intricately interwoven. It is also termed as true mixed medullary-follicular thyroid carcinoma (MMFTC). Type II(collision tumors) is characterized by distinct papillary and medullary thyroid carcinomas that come into contact and form a single mass within the thyroid gland. Type III (separate tumors in the same lobe) has the medullary and papillary thyroid carcinomas present in different areas of the same thyroid lobe, separated by normal thyroid tissue. Type IV (tumors in different lobes) has medullary and papillary thyroid carcinomas located in separate thyroid lobes or in the isthmus [6-8].

The MMFTC is characterized by features of both MTC and follicular-derived carcinomas (eg, papillary or follicular thyroid cancer). MMFTC can occur in three ways: like MTC and follicular cell hyperplasia, MTC and PTC, and MTC and DTC with cells having positivity for thyroglobulin as well as calcitonin [9].

The mathogenesis of this coexistence remains unclear. The "collision theory" assumes that the coexistence of two histologically distinct thyroid malignancies, arising independently within the same thyroid gland, is coincidental. They collide anatomically due to their close physical proximity within the thyroid gland. Pathological examination of collision tumors shows clear boundaries between the two tumor types, and they retain their distinct genetic and phenotypic characteristics. The "field effect theory" states that the entire thyroid gland is exposed to a common genetic, environmental, or a common carcinogenic stimulus (e.g., radiation exposure, smoking) that can lead to synchronous tumorigenesis. The "stem cell theory" suggests that a common multipotent progenitor stem cell in the thyroid gland undergoes divergent differentiation along different cellular lineages, giving rise to distinct tumor types. The "common pathway activation" leads to activation of shared molecular pathways or common molecular mutations leading to synchronous tumors [10-15]. Another possible theory is the "hostage hypothesis", which suggests that the malignant transformation of parafollicular C cells, along with the entrapment of normal follicles, creates a microenvironment that promotes the neoplastic growth of the trapped follicular cells [16].

In our case, though FNAC was Bethesda category III, she had raised serum calcitonin and CEA in a background of strong family history of MTC (RET mutated), we proceeded with total thyroidectomy with prophylactic central and bilateral level II to V neck dissection. The final histopathology revealed both MTC and PTC in the same thyroid gland but in different lobes with intervening normal thyroid parenchyma. Our case was Type IV synchronous multiple MTC-PTC with papillary microcarcinoma. In a series of 82 thyroidectomies performed for medullary thyroid carcinoma, 14.7% of cases showed the presence of both medullary and papillary thyroid cancers occurring simultaneously in the same thyroid gland [17].

Our patient also had a central compartment neck node that showed tumor deposits from medullary and papillary carcinoma, which was confirmed by immunohistochemistry (CK19 strongly positive, CD56 faint positivity, and synaptophysin positive). This is an extremely rare phenomenon for a single node to harbor metastatic deposits from both tumors. PTC and MTC could metastasize independently to the same lymph node due to the shared lymphatic drainage pathways. This coexistence in a single node may be purely coincidental ("collision theory"). To our knowledge, ours is one of the very few cases reported in literature. We found only eight such case reports with synchronous MTC and PTC featuring collision nodal metastasis within the same lymph node [18-24] (Table 2).

**Table 2:** Reported cases of synchronous MTC and PTC with nodal metastasis to the same lymph node MTC - medullary thyroid cancer; PTC - papillary thyroid cancer

Case number	Year of publication	Author	Country	Demographics	Procedure	Pathology findings of thyroid	Pathology findings in node
1	1996	Pastolero et al. [18]	Canada	41 years, male	First surgery - Left lobectomy with excision of medial part of right lobe + level VI neck dissection; Second surgery - completion thyroidectomy	PTC-subcentimetric, MTC -2.5cm	Combined metastasis in three nodes
2	2003	Papi et al. [19]	Italy	72 years, female	Total thyroidectomy with bilateral and central neck dissection	PTC- subcentimetric, MTC (bilateral)- 2.2 cm	Combined metastasis in level VI
3	2004	Seki et al. [20]	Japan	36 years, male	First surgery - total thyroidectomy + neck dissection; Second surgery - revision neck dissection	PTC-subcentimetric, MTC-6.5 cm	Combined metastasis - level unknown
4	2004	Seki et al. [20]	Japan	78 years, female	Right lobectomy + neck dissection	PTC-subcentimteric, MTC- 6 cm	Combined metastasis in level VI
5	2004	Meshikhes et al. [21]	Saudi Arabia	39 years, male	Total thyroidectomy with central compartment neck dissection	PTC- 2cm, Multifocal MTC- 1.5 cm	Combined metastasis in level VI
6	2005	Nicolas et al. [22]	USA	67 years, male	First surgery - Subtotal thyroidectomy; Second surgery - Completion thyroidectomy + Bilateral neck dissection	PTC- 1.2 cm, Bilateral MTC- subcentimetric	Combined metastasis in two nodes , location unspecified
7	2011	Sadat Alavi et al. [23]	Iran	32 years, male	Total thyroidectomy with central compartment neck dissection	PTC- 1 cm MTC- 3cm	Combined metastasis in one node , location unspecified
8	2020	Li et al. [24]	Singapore	27 Years, male	First surgery - Total thyroidectomy + left paratracheal node dissection + left lateral neck dissection; Second surgery - revision central compartment neck dissection	Multifocal PTC and multifocal MTC	Combined metastasis in two nodes (para tracheal); In second surgery - 3 out of 7 nodes had metastasis. All the 3 nodes showed combined metastasis

Surgery is the standard of care treatment for thyroid cancer. The coexistence of MTC and PTC in the same thyroid gland is rare and is often an incidental finding in histopathology. There are no unified guidelines or expert consensus on the appropriate extent of surgical management for this condition. The American Thyroid Association (ATA) guidelines recommend radical surgery in patients with both MTC and PTC, as MTC is more aggressive and requires a more extensive surgical approach compared to PTC. Surgical management of MTC is always total thyroidectomy with central compartment neck dissection. The need for lateral neck node dissection is based on the imaging and serum calcitonin levels.

A comprehensive surveillance with a long-term follow-up to monitor for recurrence or metastasis of both tumor types is mandatory. This includes regular clinical examination and serum calcitonin and carcinoembryonic antigen (CEA) levels for MTC, as well as serum thyroglobulin (Tg) and anti-thyroglobulin antibody levels for PTC with ultrasound of the neck. A rise in serum tumor markers may warrant additional imaging. The occurrence of PTC and MTC is often due to BRAFV600E and RET proto-oncogene mutations, respectively. Abnormal expression of the RET gene is also observed in 20-40% of papillary thyroid carcinomas. RET mutation involving exons 11 and 16, which are frequently linked to MEN 2A and MEN 2B, respectively, are strongly associated with a higher risk of lymph node metastasis, and particularly the exon 16 mutation has a very aggressive course. In this patient, a BRAFV600E and RET mutation study was not done. However, it is equally important to carry out genetic testing in such patients to confirm syndromic association in view of multiple gene mutations, and also screen for other malignancies that can occur because of such mutations.

Our patient was advised to undergo radioactive iodine therapy after our institute's multidisciplinary tumor board consensus. She completed her first postoperative follow-up at one month, during which serum calcitonin and carcinoembryonic antigen (CEA) levels were within normal limits, and the unstimulated serum thyroglobulin (Tg) was less than 0.2 ng/mL (Table 3).

**Table 3:** Postoperative serum tumor markers

Serum markers	Result	Reference value
Serum calcitonin	4.8 pg/ml	0-11.50 pg/ml
Serum carcinoembryonic antigen (CEA)	2.5 ng/ml	Non smoker : <3.8 ng/ml ; Smoker : <5.5 ng/ml
Unstimulated serum thyroglobulin (Tg)	<0.2 ng/ml	1.50-38.5 ng/ml

PTC generally has an excellent prognosis, while MTC is more aggressive, especially in advanced or metastatic settings. The 10-year disease-free survival rate for non-metastatic synchronous MTC-PTC was 87%, which was substantially higher than the 81% observed for isolated MTC, and also patients with synchronous MTC-PTC had a slightly better prognosis than MTC alone [25]. As per studies, most of the deaths in MTC-PTC tumors were reported to be due to distant metastasis due to the MTC component.

Options of adjuvant treatment in such synchronous tumors include radioactive iodine therapy, endocrine therapy, and targeted drug therapy like tyrosine kinase inhibitors and selective RET inhibitors for RET mutant MTC, based on the stage of the disease. Data on radiation and chemotherapy are still being evaluated [26].

## Conclusions

Synchronous PTC-MTC tumors are rare and often diagnosed incidentally in histopathology. Accurate diagnosis requires thorough histopathological analysis, extensive sampling, and the use of immunohistochemical markers and molecular testing. Failing to identify the MTC component may lead to inadequate surgical management, including insufficient lymph node dissection and a lack of postoperative monitoring of serum calcitonin and CEA. Similarly, missing PTC could result in the omission of necessary adjuvant radioactive iodine therapy. These dual pathologies underscore the importance of comprehensive genetic and molecular analysis, as shared mutations like RET or BRAF may drive their concurrent occurrence and be associated with other cancers. Long-term follow-up with clinical examination, tumor markers, and imaging is essential. Further molecular research is needed to understand the pathogenesis of these synchronous tumors, offering insights into genetic, epigenetic, and micro-environmental factors to improve diagnosis and treatment strategies.
